# Congenital adrenal hyperplasia presented with bilateral testicular tumor: A case report

**DOI:** 10.22088/cjim.12.0.431

**Published:** 2021

**Authors:** Abazar Akbarzadeh Pasha, Hamid Shafi, Mohamad Teimorian, Ghasem Rostami, Khatereh Nasirimehr, Emadoddin Moudi

**Affiliations:** 1 Cancer Research Center, Health Research Institute, Babol University of Medical Sciences, Babol, Iran; 2 Clinical Research Development Center, Shahid Beheshti Hospital, Babol University of Medical Sciences, Babol, I.R.Iran.; 3 Department of Urology, Shahid Beheshti Hospital, Babol University of Medical Sciences, Babol, Iran; 4 Student Research Committee, Babol University of Medical Sciences, Babol, Iran

**Keywords:** Congenital adrenal hyperplasia, Adrenal rest tumor, Leydig cell tumor

## Abstract

**Background:**

Congenital adrenal hyperplasia (CAH) refers to group of congenital diseases resulting from impaired adrenal steroidogenesis, and its most common cause is 21-hydroxylase deficiency. Testicular adrenal residual tumor (TART) is one of the major complications of CAH, possibly resulting from ectopic remnants of intra-testicular adrenal tissue which is stimulated by excessive secretion of adrenocorticotropic hormone (ACTH). This tumor can be misdiagnosed as Leydig cell tumor (LCT) in these people.

**Case Presentation:**

The patient we are presenting is a 20-year-old man with a history of precocious puberty and a height below 3% of the population who underwent radical left testicular orchiectomy with a complaint of bilateral testicular mass, which is reported LCT in the pathology report. In preoperative imaging examinations, bilateral adrenal hyperplasia is observed. In hormonal examinations, the patient is diagnosed with CAH and has been treated with corticosteroids for one year.

**Conclusion:**

In patients who present with bilateral testicular mass, it is the best image by abdominopelvic CT scan before surgery to detect CAH.

Congenital adrenal hyperplasia (CAH) refers to group of congenital diseases resulting from impaired adrenal steroidogenesis, and its most common cause is 21-hydroxylase deficiency. Testicular adrenal residual tumor (TART) is one of the major complications of CAH, possibly resulting from ectopic remnants of intra-testicular adrenal tissue which is stimulated by excessive secretion of adrenocorticotropic hormone (ACTH). This tumor can be misdiagnosed as Leydig cell tumor (LCT) in these people. Due to the possibility of histological misdiagnosis between TART and LCT, this case was reported to avoid unnecessary with TART diagnosis in mind in similar patients. 

## Case Presentation

The patient is a 20-year-old man who noticed a swollen testis about a year ago after falling off a motorcycle, which was painful but did not follow-up due to the lack of continued pain. A year later, he referred to a urologist again, who on examination, noticed swelling and a mass in both testes, and requested ultrasound and tumor markers for the patient. On ultrasound, a 26 × 21 × 26 mm hypoechoic mass containing echogenic foci suggesting calcification in the left testicle and another hypoechoic foci measuring 8 ×8 × 8 mm in the lower pole of the right testis and another small foci of 2 mm diameter was observed in the upper pole of the same testis, which primarily indicated a testicular tumor. In measuring tumor markers, BHCG, AFP and LDH markers were within the normal range.

 Due to the ultrasound and larger and painful left testicular mass, he became a candidate for left orchiectomy. In preoperation examinations, ultrasound was performed again and then computed tomography (CT) scan was requested for the patient. Complete abdominopelvic ultrasound before operation, reported bilateral adrenal hyperplasia was observed which eventually was recommended to perform CT scan. Examination of the testicles showed diffuse microlithiasis in both testes. At least two masses were seen in the left testis, the largest of which was 27 × 27 mm hypoechoic containing calcified foci with vascular flow. At least 4 hypoechoic masses with vascular flow were observed in the right testis, the largest of which was 5 × 3 mm. CT scan showed bilateral adrenal hyperplasia and two lymph nodes with maximum SAD of 7 mm in the para-aorta and aortocaval.

The patient underwent a radical left orchiectomy and the pathology of removed testis reported consistently with sex cord stromal tumor in favor of Leydig cell tumor and in immunohistochemically (IHC) evaluation, calretinin was positive and CD99, WT1 and Melan A were weakly positive. After the operation, due to adrenal hyperplasia, hormonal tests were requested and the patient was referred to the endocrinology outpatient clinic. The patient has no history of hospitalization in infancy. He states that he was taller than his peers at the age of 7, but he has not increased in height compared to his peers since he was 10 years old. He did not mention the history of allergies to drugs and food and any sort of drug consumption. The parents were not related and his brother died at a young age (possibly a stroke). On examination of the patient, hair growth was sufficient and had a masculine pattern. He did not have gynecomastia on chest examination. 

The patient did not have a history of hypertension and on examination, the patient's blood pressure was normal. The patient is 154 cm tall and weighs 60 kg. The patient's BMI was measured at 25.2kg/m^2^. Due to the presence of bilateral adrenal hyperplasia in the imaging, the patient underwent laboratory tests due to suspicion of congenital adrenal hyperplasia. The laboratory tests showed high levels of aldosterone (1234, 1 pg/mL, 30-400), ACTH (219 pg/mL, 5-46), Renin activity (131,8 u/mL, 4,4-46,1) fasting ACTH (179,7 pg/mL, 5-55), plasma renin activity (10, 2 ng/mL/hr , 0,06-4,3), and 17-OH progesterone (2520 ng/mL , 5-30) and then due to the patient's age, adrenal hyperplasia on CT scan, history of precocious puberty and high levels of ACTH, the patient was diagnosed with CAH. Patient was then diagnosed with TART and was treated with corticosteroids for one year, which reduced the number of right testicular masses. The patient is still in the follow-up period.

**Figure 1:**
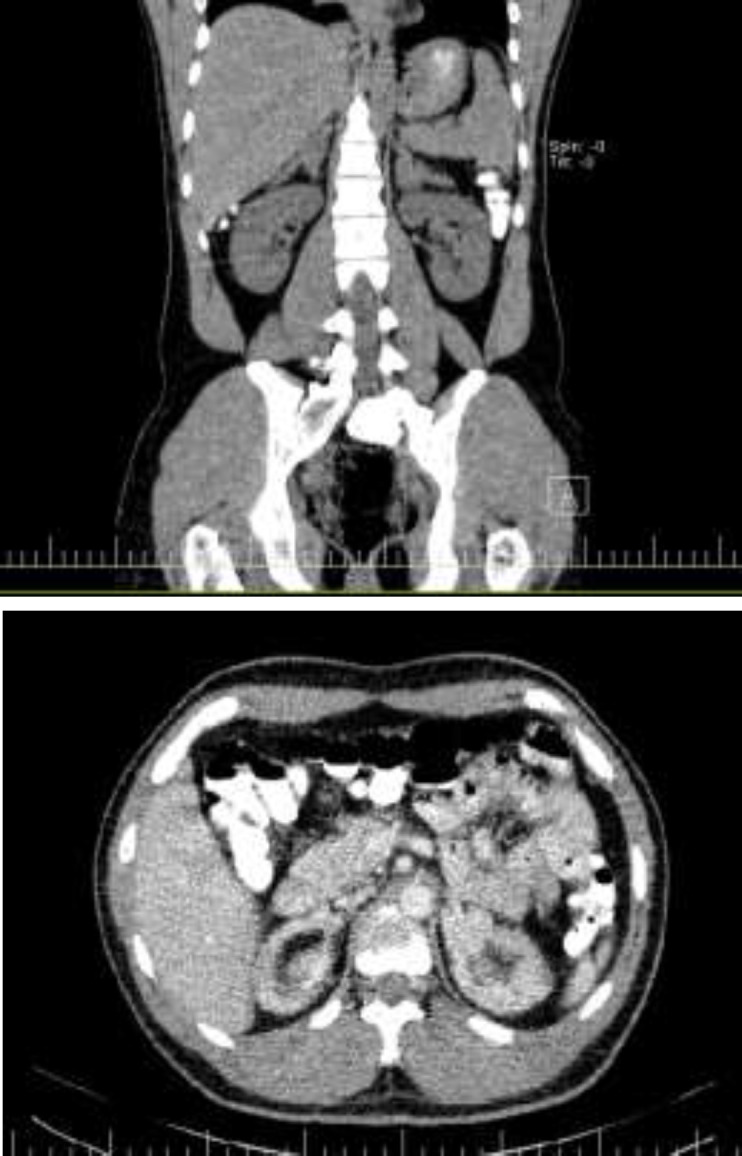
CT scan imaging of patient before left orchiectomy in coronal (a) and transverse (b) sections

## Discussion

Congenital adrenal hyperplasia is one of the most common autosomal recessive genetic disorders which caused by CYP21 (21-hydroxylase) deficiency in more than 90% of patients (1). Its prevalence is approximately 1 in 15,000 births. In people with CAH, the production of cortisol and aldosterone is disrupted, leading to increased production of ACTH by the pituitary gland. Excessive amounts of ACTH cause adrenal hyperplasia and overproduction of adrenal androgens (2). 

Testicular lesions in CAH were first described by Wilkins et al. (3). These lesions are commonly known as testicular adrenal rest tumor or TART. The hypothesis for TART is that there are abnormal adrenal cells that go down with the testes during embryogenesis. Most TARTs are small and not clinically detectable, but can rarely appear as a palpable clinical mass (4). Residual ectopic adrenal tissue is found in retroperitoneum, ovaries, inguinal, round ligament, and testes of 50% of normal neonates (5). Typically, this ectopic tissue becomes atrophic during growth and remains in less than 1% of individuals. In patients with CAH, TART occurs due to adrenal hyperplasia with overstimulation of ACTH (6). In most cases, the diagnosis of CAH is made before the diagnosis of TART. However, due to the wide range of types and severity of the disease, the clinical manifestations are different. Rarely, mild forms of the disease may not develop until early adulthood (5).

 TART is seen in 18% of patients with undiagnosed CAH and may be the first symptom of that (6). TARTs are most commonly seen in adolescence and early adulthood because elevated LH levels can stimulate tumor growth during these periods (7). 

Although TARTs are benign, they can be mistaken for Leydig cell tumors (LCTs). With a misdiagnosis, the patient undergoes unnecessary orchiectomy, whereas TARTs typically respond to corticosteroid therapy and do not require surgery. Because of that, it is necessary to distinguish between these two. Correct diagnosis of TART from LCT is possible through thorough imaging, clinical, and histological examinations. While both LCT and TART occur in early adulthood (9), TART is typically associated with CAH. Biochemical evaluation of hormone profiles (17-OH-progesterone, 11-deoxycortisol, dehydroepiandrosterone, and androstenedione) is useful for CAH confirmation. In addition, TARTs are bilateral in more than 80% of cases, while LCT is bilateral in only 3%. Ultrasound is a good but non-specific way to diagnose TART and follow-up (10). 

In addition, bilateral LCT and the concomitant presence of TART and LCT in patients with CAH have also been reported in studies (15, 16). TART is usually located in the rete testis and can grow to cover the entire testes. They form single or multiple dense nodules or irregular nodular masses. In sections, they form multilobulated light brown foci which were separated by narrow greyish bands of fibrous tissue (11). 

Histologically, TARTs are similar to adrenal cortex tissue (12). Large polygonal cells with abundant eosinophilic cytoplasm are arranged in strands or cords. Features that are more common to TART compared with LCT include lack of cytological atypia, low mitotic activity, dense fibrous septa, lymphoid aggregates, adipose metaplasia and prominent lipochrome pigment. Reinke crystals, which can be found in 25-40% of LCTs, are absent in TART. Immunohistochemically, TART shows diffuse and strong positivity for CD56, focal or diffuse strong reactivity for synaptophysin and negative reactivity for the androgen receptor. In contrast, LCT displays focal weak to moderate or negative reactivity for CD56 and focal weak or negative reactivity for synaptophysin, but positive reactivity for the androgen receptor (13, 14). 

Imaging is essential in the diagnosis and follow-up of TARTs. The first imaging is ultrasound which is a convenient and cost-effective method. Many of these lesions are hypoechoic and have a definite margin, but large lesions can be hyperechoic. On Doppler ultrasound, these lesions are usually hypervascular (17). MRI is not completely confirmed. Some studies report that TART margins can be well detected on MRI. When planning for surgery, MRI is preferable to ultrasound in describing the extent and margin of TART (18). In T1 MRI imaging, TARTs are usually iso-intense, whereas in T2, they are hyper intense (19). To date, high dose glucocorticoid therapy has been the treatment of choice in patients with TART, which may reduce tumor size by suppressing ACTH secretion, thereby improving testicular function (20). 

In conclusion In this case, the patient presented with a painless bilateral testicular mass of unknown origin who underwent unilateral orchidectomy due to the malignant appearance on ultrasonography of the testes. In this case, it was difficult to differentiate TART from LCT, the main reason for that is the lack of CAH diagnosis in the patient's history. 

On the other hand, in the ultrasound, the masses were hypoechoic and despite the fact that the patient did not know that he has CAH, malignancy was at the top of our list of differential diagnoses. Another subject is the patient's pathology report, in which due to the similarity of the histological features of TART with LCT, in the absence of diagnostic suspicion of TART, is easily mistaken and leads to misdiagnosis. Immunohistochemical examination, especially the response of CD56, synaptophysin and CD10 can be helpful in the diagnosis of TART. 

According to the case, it is recommended that TART be part of our differential diagnosis in all people with CAH who present with testicular tumors, especially bilateral tumors. In cases where the patient presents without history of CAH and with bilateral tumor, it is best to perform an abdominal CT scan to examine the patient's adrenals before orchiectomy.
